# The Security of IP-Based Video Surveillance Systems

**DOI:** 10.3390/s20174806

**Published:** 2020-08-26

**Authors:** Naor Kalbo, Yisroel Mirsky, Asaf Shabtai, Yuval Elovici

**Affiliations:** 1 Software and Information Systems Engineering, Ben-Gurion University of the Negev, Beer Sheva 8410501, Israel; kalbo@post.bgu.ac.il (N.K.); shabtaia@bgu.ac.il (A.S.); elovici@bgu.ac.il (Y.E.); 2 College of Computing, Georgia Institute of Technology (Georgia Tech), Atlanta, GA 30332, USA

**Keywords:** IP cameras, video surveillance, IoT, network security, physical security

## Abstract

Over the last decade, video surveillance systems have become a part of the Internet of Things (IoT). These IP-based surveillance systems now protect industrial facilities, railways, gas stations, and even one’s own home. Unfortunately, like other IoT systems, there are inherent security risks which can lead to significant violations of a user’s privacy. In this review, we explore the attack surface of modern surveillance systems and enumerate the various ways they can be compromised with real examples. We also identify the threat agents, their attack goals, attack vectors, and the resulting consequences of successful attacks. Finally, we present current countermeasures and best practices and discuss the threat horizon. The purpose of this review is to provide researchers and engineers with a better understanding of a modern surveillance systems’ security, to harden existing systems and develop improved security solutions.

## 1. Introduction

These days, video surveillance systems can be found everywhere. They are in the streets, at train stations, workplaces, factories, and even at home. Intelligent applications have made large surveillance networks practical to manage and utilize. For example, technology for facial recognition, identifying threats, event-detection, tracking objects, and rapidly investigating incidents can be scaled to thousands of cameras over large geographical areas.

Over the last few decades, surveillance technologies have evolved from analog systems to packet switched systems (over IPv4 & IPv6 networks) [1]. Analog systems use cameras that transmit analog signals (typically over a coax cables) to a recording unit. Attacks on these systems were either denial of service by cutting wires, video injection by intercepting the cable, and record tampering by physically interacting with the recording unit. In contrast, IP-based systems have diverse topologies and technologies making them far more complex and which results in much larger attack surface (e.g., VPNs, gateways, multiple servers, WiFi, access control systems, etc.). Furthermore, these systems are often exposed to the Internet enabling attackers to continuously attack them while new vulnerabilities are discovered everyday.

Regardless, IP-based video surveillance systems have become affordable due to the popular and pervasive Internet of Things (IoT). As a result, the market for security devices in connected homes has grown by a factor of 17 over the last few years [2]. Due to their convince, practicality, and affordability, video surveillance systems have become ubiquitous in our daily lives.

However, as expected, these systems and their components have been the target of numerous cyber attacks. For example, they have been the target of distributed denial of service (DDoS) attacks, exploited to invade the users’ privacy, and even to mine cyrpto-currency [3]. These systems have also been recruited into botnets to perform nefarious tasks. For example, in 2014, the infamous Mira botnet targeted surveillance systems, and infected over 600,000 devices worldwide [4].

Through Shodan.io and Censys.io queries of well known manufacturers, we found over 1 million surveillance cameras and over 125,000 surveillance servers exposed to the Internet. Of these devices, 90% do not have secure login portals (use HTTP and not HTTPS). Moreover, approximately 8% have open SSH and Telnet ports, 3% have exposed MySQL databases, and at least 1.7% of these devices are still vulnerable to the HeartBleed SSL vulnerability discovered in 2012. Even large video surveillance manufacturers have exposed products. For example, Samsung’s CCTV Server has at least 83,035 exposed devices, where 86% of them use HTTP login portals, and 1604 have ssh ports open. Moreover, HikVision, the surveillance manufacturer with the largest market share of 24.7% has at least 260,415 exposed devices where only 53 of them had HTTPS enabled, but with self-signed certificates. In one study, it was found that approximately 73,000 security cameras in 256 countries are accessible with default passwords [5]. These statistics emphasize the poor state of security of IP-based surveillance systems. Moreover, these systems are highly targeted. In [6], the authors showed how their surveillance system was hacked after two minutes of being connected to the Internet.

In this article, we will review the cyber security of modern surveillance systems. We will start by detailing the composition and topology of modern video surveillance systems. Next, we will understand the goals of an attacker by discussing them in terms of their effect on the confidentiality, integrity, and availability of the system. Afterwards, we will explore how an attacker can realize his/her goal through multiple attack steps involving different threat agents and malicious actions. We will exemplify these attacks with current events and published common vulnerability exposures (CVE). Finally, we will review best practices and known security solutions which can be used to help mitigate these cyber threats.

To capture the attack surface, we conducted our review as follows: (1) we identified the relevant elements (assets and actors) using real-world deployments, (2) we searched for relevant academic papers and technical reports relating to attacks and countermeasures for each element and these systems in general, (3) we searched Shodan to identify the relevant threats, (4) we executed existing IP-camera attacks (such as video injection) to enumerate and understand the system’s limitations, and (5) we used penetration testers to search for attack vectors and novel vulnerabilities in a real commercial surveillance system used in seaports, airports, railways, and in other commercial zones. Details on the testbed’s configuration can be found in Section 2.5.

We note that many other works have performed comprehensive reviews of IoT security [7,8,9]. Although IP-based surveillance systems use generic network elements and share technologies with other IoT systems, their attack surface is different from a cybersecurity stand-point. This is because they support and enforce our physical security, and, when compromised, there is a threat to our physical safety and our privacy (at home, at work, and in a national sense). As a result, the technology, threat actors, and attack motivations, and overall attack surface differ from other networks and IoTs. We distinguish these differences as follows:

**Threat Actors** **(Who)** There is a set of attackers who want to exploit the functionality of surveillance systems specifically. For example, state-actors or thieves performing reconnaissance over a geographic area and criminals planning to blackmail a victim with video footage.
**Assets** **(What)** If compromised, these systems can provide an attacker with private imagery resulting in a direct explicit violation of privacy. These systems are also lucrative assets to botnet owners since they typically have high bandwidth (for DDoS attacks) and decent compute capabilities (for cryptomining). The features of a surveillance system change the weight of attacker’s goals and the defender’s priority on the defenses. For example, there is more emphasis on anti-DoS and MitM attacks in surveillance systems than other systems. Overall, the privacy violation of exposed data has much stronger implications than data from other IoTs.
**Topology** **(Where)** Unlike other IoTs, surveillance systems are often centralized systems connected to a single server. They are also commonly connected to both the Internet and an internal private network—thus exposing a potential infiltration vector. It is also common for surveillance systems to have their server physically on-site, as opposed to being in the cloud. These aspects change attack surface by encouraging the attacker to find other ways to compromise the system (e.g., via local attacks).
**Motivation** **(Why)** Aside from being a stepping stone into another network, surveillance systems elicit monetary motivations such as blackmail, cryptomining, and spying for military or political reasons. Moreover, an attacker can have a physical advantage if the system is targeted in a DoS attack. For example, stopping video-feeds in certain geographic areas prior to an attack/theft, or as an act of cyber terrorism. Another aspect to consider is that a DoS attack on a generic IoT device or information system is a nuisance, whereas on a surveillance system it means that the attacker can remotely disable video feeds at will. For example, a VPN router in the surveillance system can be targeted remotely causing a loss of signal to all cameras.
**Attack Vectors** **(How)** Surveillance systems have unique security flaws and attacks due to their functionality. Some examples include (1) servers which accept self-signed certificates (which can lead to man-in-the-middle attacks) just to be compatible with many different camera models of various vendors, (2) their unique susceptibility to side channel attacks on encrypted traffic due to the nature of video compression algorithms, (3) video injection attacks where a clip of footage is played back in a loop to cover up a crime, and (4) data exfiltration performed via a camera’s infrared nigh vision sensor. Moreover, modern systems rely on machine learning algorithms to identify and track objects and people. Unlike AI on other IoTs, these AI models can be easily evaded/exploited due to their accessibly and flaws [10].

Although many components of IP-based surveillance systems are similar to those of other IoTs from a technological perspective (e.g., the camera and smart fridge can both have vulnerable telnet portals), it is much more important to identify the attacker’s motivation (target assets) and strategies (e.g., how to infiltrate and be covert) in order to design safe architectures and effective defenses. Thus the attack surface is not the culmination of the individual software and hardware vulnerabilities, but rather the attacker’s intent and motivation which drive the attack goals and strategies. Knowing the goals and strategies are key to developing a secure system and designing an effective countermeasure.

We also note that here is a good security review of CCTV and Video Surveillance Systems in [11]. However, in [11], the authors focus on visual attacks such as data exfiltration, covert channels, and steganography. The objective of this review is to provide the reader with a deeper understanding of an IP-based Surveillance System’s attack surface so that he or she may better understand the attacker’s goals and techniques in the context of these systems.

The structure of this review is as follows. In Section 2, we present a system overview with a security oriented taxonomy, a description of the various targets (assets), and the common deployment topologies. In Section 3, we detail the attacker’s possible motivations in the form of security violations. In Section 4.2, we identify the elements of attack vectors against these systems (threat agents, actions, and consequences) followed by some example attack vectors comprised of these elements. We also ground all of these attacks and vulnerabilities with real world cases. In Section 5, we discuss countermeasures and best practices for securing IP-based surveillance systems against each of the various attacks. Finally, in Section 6 we provide a discussion on the threat horizon, suggest future work, and conclude the review.

## 2. System Overview

Before we can discuss the security aspects, we must describe what a surveillance system is. In this section, we first present a general overview of IP-based video surveillance systems. Afterwards, we list some of the system’s critical assets and common deployment schemes.

### 2.1. Overview

**Figure 1:**
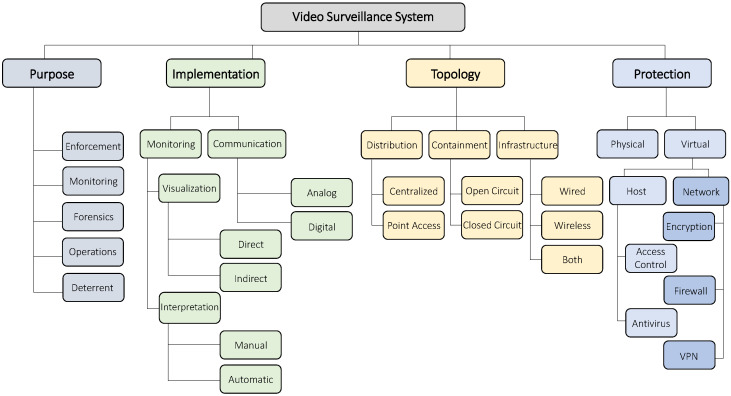
An overview of video surveillance systems.

To get a better understanding of IP-based video surveillance systems, in Figure 1, we present a taxonomy of their concepts. In general, a system can be described in terms of its purpose, implementation, topology, and protection. We will now detail each of these aspects. To create it, we started with a basic taxonomy which is based on common knowledge and our experience in cyber security. We then evolved the taxonomy based on input from surveillance system engineers, security experts, and the papers, vulnerability disclosures, and reports which we reviewed. We verified that the information could be mapped to our taxonomy, and, if not, we updated it.

**Purpose**  The purpose of a video surveillance system depends on the user’s needs.
  **Enforcement**  The user may want to send security forces or police to an area undergoing some violation of law or protocol. This is common in governments, transportation services, stores, and even workplaces.
  **Monitoring**  The user may want to know what is happening in a certain location for some general purpose, or to have a sense of security—for example, home, baby, and pet monitoring.
  **Forensics**  The user may want to be able to produce evidence or track down an individual.
  **Operations**  The user may want to improve operations by having an overview of what is going on. For example, employees can be guided or managed more efficiently.
  **Deterrent**  The user may want to have the system visually present to simply ward off potential offenders or trespassers. In some cases, the user will not even have a means of viewing the video footage.
**Implementation**  There are various ways the hardware/software of the system can be setup to collect and interpret the video footage. We categorize the system’s implementation into two categories:
  **Monitoring**  This concept regards how the user visualizes the video streams, and how the content is interpreted. The visualization can be provided directly to the user directly such as in a closed circuit monitoring station, or indirectly via a digital video recorder (DVR) with remote access or in the cloud. The interpretation of the content can be done manually by a human user reviewing the content, or automatically via motion detection, or advanced applications such as object tracking, image recognition, face-detection, and event-detection.
  **Communication**  This refers to the means in which the system transports the video feeds [1]. With analog methods, the video is sent to the DVR as an analog signal (which is subsequently connected to Internet). With digital methods, the video is processed, compressed, and then sent as a packet stream to the DVR via IPv4 and IPv6 network protocols. A common approach is to compress the stream with the H.264 codec and then send it over the network with a real-time protocol such as RTP over UDP.
**Topology**  An IP-based surveillance system’s topology can be described by its distribution, containment, and infrastructure. Distribution refers to whether the cameras are located anywhere in the world or physically located in one area. Containment refers to whether the system is closed circuit (not connected to the Internet) or open circuit—and relies on access control to deny users without proper credentials. Finally, infrastructure refers to how the elements of the system are connected together: wireless (e.g., Wi-Fi), wired (e.g., Ethernet via CAT6 cables), or both.
**Protection**  The protection of surveillance system refers to how the user secures physical and virtual access to the system’s assets and services. Without physical protection, an attacker can tamper/damage the cameras or install his/her own equipment on the network. Virtual protection can be employed on the network hosts or on the network itself:
  **Host**  Cameras, DVRs, and other devices can be protected by using proper access control mechanisms. However, like any computer, these devices are subject to the exploitation of un/known vulnerabilities in the software, hardware, or simply due to user misconfiguration (e.g., default credentials) [4]. Protecting the hosts from attacks may involve anti-virus software or other techniques.
  **Network**  Depending on the topology, access to the system’s devices may be gained via the DVR, an Internet gateway, or directly via the Internet. A user may protect the devices and the system as a whole by securing the network via encryption, firewalls, and end-to-end virtual private network connections (VPN).

### 2.2. Assets

An asset is a thing of value which may be targeted by an attacker. In our case, the assets are data, devices, software, and infrastructure:

**DVR—Media** **Server** The digital video recorder, or other media server, which is responsible for receiving, storing, managing, and viewing the recorded/archived video feeds. DVRs are typically an application running on the user’s server, or a custom hardware Linux box. DVRs can also be a cloud based server. In a small system, there may be cameras which do not support a DVR, and require the user to connect to the camera directly (e.g., via web interface).
**Cameras**  The devices which capture the video footage. There are many types, brands, and models of IP-Cameras, and each has its own capabilities, functionalities, and vulnerabilities. For configuration, some IP cameras provide web-based interfaces (HTTP, Telnet, etc.) while others connect to a server in the cloud. Most cameras act as web servers which provide video content to authorized clients (e.g., the DVR will connect to the camera as a client).
**Viewing** **Terminal** The device/application used to connect to the DVR or camera in order to view and manage the video content. For example, an Android application running on a smartphone or the DVR itself.
**Network** **Infrastructure** The elements which connect the cameras to the DVR, and DVR to the user’s viewing terminal—for example, routers, switches, cables, etc. The infrastructure also includes Virtual Private Network (VPN) equipment and links. VPNs are LANs which tunnel Layer 2 (Ethernet) traffic across the Internet, between gateways and user devices, using encryption. Site-to-site VPNs can bridge two segments of a the surveillance network over the Internet. A remote-site connection tunnels traffic directly from a user’s terminal to the surveillance network.
**Video** **Content** The video feeds which are being recorded or that have been archived for later viewing.
**User** **Credentials** The usernames, passwords, cookies, and authentication tokens used to gain access to the DVR, cameras, and routers. The credentials are used to authenticate users and determine access permissions of video content, device configurations, and other assets.
**Network** **Traffic—Data** **in** **Motion** Data being transmitted over the network infrastructure. This can be credentials, video content, system control data [12] (e.g., pan, tilt, or zoom), and other network protocols (ARP, DNS, HTTP, SSL, TCP, UDP, etc).

### 2.3. Deployments

**Figure 2:**
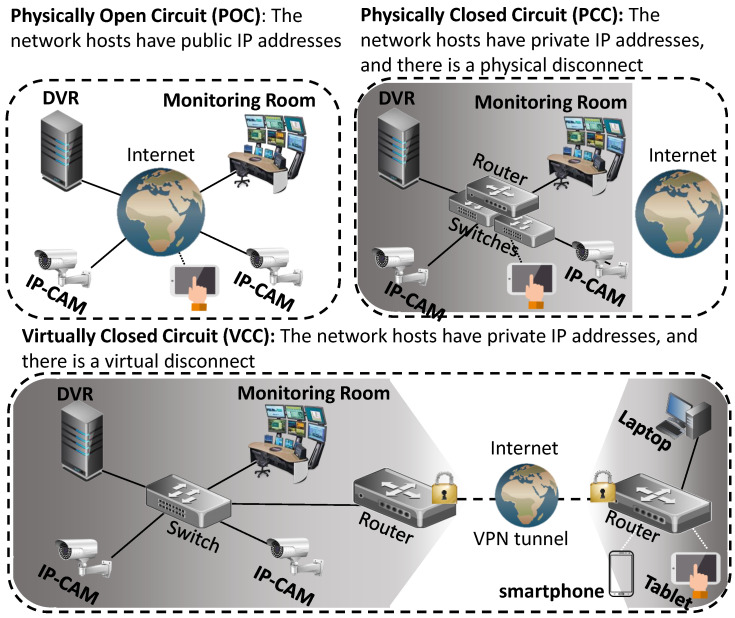
Accessibility models for deploying a video surveillance system.

There are several ways an IP-based surveillance system can be deployed. The network topologies can be centralized (all cameras connect to a DVR) or distributed (the user connects to each individual camera). In terms of accessibility, the system can be directly accessible via the Internet, or not at all. In this regard, we identify three categories of accessibility (visualized in Figure 2):

**Physically** **Open** **Circuit** **(POC)** When the network hosts in the system (cameras, DVR, etc.) have public IP addresses. This means that anybody from the Internet can send packets to the devices.
**Physically** **Closed** **Circuit** **(PCC)** When the network hosts in the system have private IP addresses, and there is no infrastructure which connects the network to the Internet. This means that noone from the Internet can send packets to the devices directly. These systems are also called air-gapped networks [13].
**Virtually** **Closed** **Circuit** **(VCC)** When the network hosts in the system have private IP addresses, and the network is connected via the Internet using a VPN. This means that noone from the Internet can send packets to the devices directly, unless they send packets via the VPN.

### 2.4. Surveillance Systems vs. IoTs

Many devices in an IP-based Surveillance System are the same as those in other IoT systems, but there exists some technological aspects which are unique to surveillance systems that can be exploited by an attacker. However, it is more important to understand the target system in the eyes of the attacker to design secure the systems and to devise effective countermeasures. Therefore, the security emphasis on the different components and the defense strategies are different in the case of surveillance systems.

#### 2.4.1. The Technological Perspective

There are two main aspects which distinguish surveillance systems from other IoT systems in a security perspective: the architecture and the use of artificial intelligence features.

**Architecture.**  Architecture: In security, we must consider the topology and relationship between components and not just the individual devices. Some elements only become vulnerable because of their relationship to others. In the case of IoTs, these devices often follow the same cloud based model, whereas surveillance systems have a variety of architectures and topologies which, if disregarded, can lead to severe vulnerabilities. For example, a camera may be safe from remote attacks because its traffic is sent over a VPN, but the DVR may be exposed because of its web accessibility. Another example is that the communication channels of the cameras (wired or wireless) are often exposed and cross over publicly accessible areas, giving the attacker more opportunities to infiltrate the network than in the case of IoTs. In this survey, we exemplify the architectures unique to surveillance systems compared to the IoT cloud model, and the strategies which attackers may use to exploit them.
**Artificial** **Intelligence.** In the case of IoTs, machine learning is often performed on the back-end for data-mining purposes. In the case of surveillance systems, there are unique attacks (e.g., all image based attacks) which enable attacks only relevant to surveillance systems (evasion, false evidence, DoS) that can lead to significant breaches in safety, privacy, and justice –unlike other IoTs. For example, an attacker can use adversarial markings on their clothes to evade detection, delay investigations (plant false triggers), and identify as a legitimate personnel. Recently researchers have shown how attackers can craft images which incur significant overhead on a model, causing the device to slow down in an effective DoS attack [14]. These attacks uniquely affect surveillance systems since the AI features provide attackers with new vectors towards accomplishing their goals and undermining the purpose of the system.

#### 2.4.2. The Attack Surface Perspective

The main goal of this survey is to provide researchers and engineers a clear view of the surveillance system’s attack surface by identifying how, where, and why an attacker would target it. In cyber security, these aspects are critical because all computer systems have vulnerabilities, and therefore defenders must prioritize their efforts accordingly. Concretely, if one were to approach the security of an IP-based surveillance system the same way as a typical IoT system, then the critical assets of the system would be exposed leading to significant damage.

To better understand the difference between our systems and other IoTs, let’s look at some contrasting examples:

**How.**  *Consider a DoS attack.* The classic way of an attacker performing this attack on IoTs would be to overload the device with traffic. Doing so would alert the local authorities to the camera’s area because the feed would go down. However, here the attacker is clearly motivated to be covert as to not alert the authorities to his or her presence. Therefore, the DoS would likely be in the form of (1) looping video content, (2) freezing the current frame, (3) targeting the DVR to manipulate the content since the DVR may be more exposed by its web portal, or (4) using an adversarial machine learning attack to evade detection (e.g., hide the fact that there is a person in the scene though use of an adversarial sticker).
  Another consideration is that IP-cameras generate massive amounts of network traffic compared to other IoTs. This makes it very easy to detect disruptions from attacks, this challenging the attacker to compromise the devices in other ways (e.g., prefer obtaining credentials than rooting the device).
**Where**.  *Consider a data theft attack.* The classic way of performing this attack would be through remote social engineering attacks (e.g., phishing) to access credentials or to exploit vulnerabilities directly in the IoT device itself to gain privileged access. However, in a surveillance system that is completely VPN-based, remotely targeting the device is not possible and the social engineering attacks may not provide access (e.g., in the case of time-based rotating keys). Rather, it is more likely that the attacker will perform a physical social engineering attack (e.g., plant a USB drive) or physically tamper with the network infrastructure to plant a man in the middle device. Distinguishing the difference is critical since it will not only indicate where emphasis in security design must be made but also determine where countermeasures should be placed (e.g., placing an NIDS by the cameras and not just at the web portal or DVR) and emphasized (e.g., to bury and lock up cables, even in ‘private’ hallways inside the building).
**Why.**  *Consider how the system could be leveraged.* An IoT device may be targeted for the information or resources in which it contains. However, there is more risk for a camera to be compromised for its capabilities (observing the video footage) or to act as a stepping stone in a grander attack. For the latter case, consider data exfiltration: cameras may be used to exfiltrate data past an advanced firewall since the cameras are always sending large amounts of data in real time. Here the attacker can ride this current undetected (below the noise floor) where in contrast to other IoTs, the traffic is typically sparse and easier to model. Another case to consider is the fact that IP-Cameras produce far more traffic (bandwidth) than other IoTs and therefore may be targeted for the purpose of creating massive DDoS attacks.
  We note that the risk of an attack on an IP-surveillance system is higher than other categories of IoT because (1) they are wide spread, (2) provide high payoff for the attacker (provide the attacker with stronger processors than other IoTs for bitcoin mining etc, and direct access to confidential information), and (3) directly impact the safety, privacy, and integrity (e.g., planting false evidence) of the user. Therefore, understanding the ‘why’ of the attack enables the defender to know where time and resources should be put into hardening the architecture and prevent poor design choices in topology and access control.

In summary, knowing where the attacker will target first gives an early warning and enables the defenders to purge the attackers from the system before significant damage is done. This can only be accomplished by fully understanding the attack surface from an attack motivational perspective. Therefore, we believe that this survey will contribute greatly to the researchers and engineers who read it. The how, where and why of the potential attacks are different than other IoTs, and therefore it is critical that this survey focuses on the narrative on these aspects.

### 2.5. The Surveillance System Testbed

**Figure 3:**
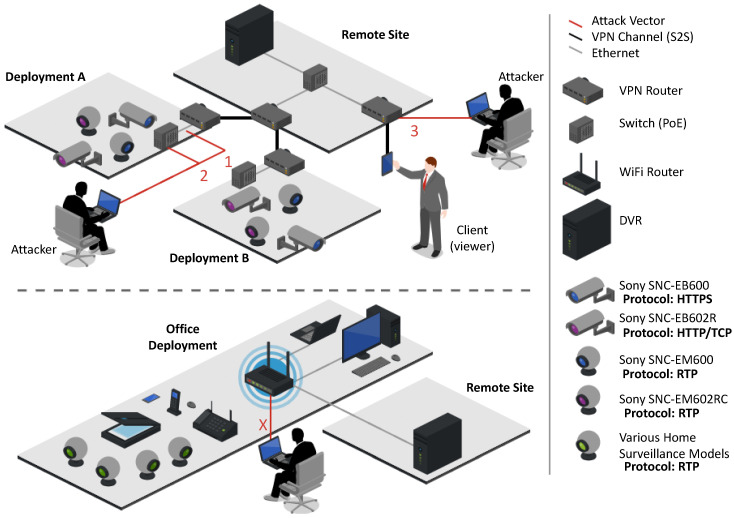
The two deployments do the commercial Surveillance System used for penetration testing during our study.

In order to identify all of the assets and attack vectors, we implemented two commercial surveillance system deployments and attacked them with two skilled penetration testers. The deployments were setup in cooperation with the supplier who also assisted in analyzing the attack vectors. Figure 3 presetns the two deployments.

In order to understand the limitations and vulnerabilities of the system, we verified nine attacks relating to different steps of a complete attack vector: reconnaissance, man-in-the-middle, denial of service (DoS) and the spread of a botnet malware. Table 1 details each of the nine attacks successfully performed and their attack vector entry points (indicated in Figure 3).

Our observations were used to complete the following sections which enumerate and identify the vulnerabilities, attack vectors, and countermeasures applicable to POC, PCC, and VCC surveillance system deployments.

## 3. Security Violations

A security violation can be described as an attack on the system’s confidentiality, integrity and availability (known as the *CIA* triad [15]). A violation of confidentiality refers to the unauthorized access of information. A violation of integrity refers to the intentional manipulation and alteration of information. Finally, a violation of availability refers to the act of preventing authorized users from accessing services or resources when needed.

The goals which an attacker may have in assaulting the system can be described in terms of the CIA triad:

1. **Confidentiality Violation**—the unauthorized access of video content, user credentials, network traffic. In this case, the attacker intends to observe the video footage for his/her own nefarious purposes. As a result, this goal puts the privacy and physical security of the premises at risk.
2. **Integrity Violation**—the manipulation of video content, or the active interference of a secure channels in the system (e.g., the POODLE SSL downgrade attack). In this case, the attacker intends to alter the video content (at rest or in transit). Alteration can include freezing frame, looping an archived clip, or inserting some other content. This misinformation can lead to physical harm or theft.
  An attacker may violate a system’s integrity for a goal which is not directly related to the video content. For example, the attacker may want to exploit the system’s vulnerabilities to gain *lateral movement* to external assets. The system may be used as a stepping stone to gain access to the following external assets:
  (a) **Internal network**—surveillance systems (especially closed circuit systems) may be connected to the organization’s internal network for management purposes. An attacker may leverage this link in order to gain access to the organization’s internal assets.
  (b) **Users**—users of the system may be targeted by the attacker. For example, the attacker may wish to install ransomware on the viewing terminal, or to hijack a user’s personal accounts.
  (c) **Recruiting a Botnet**—A ‘bot’ is an automated process running on a compromised computer which receives commands from a hacker via a command and control (C & C) server. A collection of bots is reffed to as a botnet, and is commonly used for launching DDoS attacks, mining crypto currencies, manipulating online services, and performing other malicious activities. An example botnet which infected affected IP-cameras and DVRs was the Mirai malware botnet. In 2016, the Mirai botnet generated a 1.1Tbps DDoS attack against websites, webhosts, and service providers. Another example is a worm named Linux.Darlloz which targets vulnerable devices and exploits them through a PHP vulnerability (CVE-2012-1823) [16].
3. **Availability Violation**—the denial of access to stored or live video feeds. In this case, the attacker’s goal is to (1) disable one or more camera feeds (hide activity), (2) delete stored video content (remove evidence), or (3) launch a ransomware attack (earn money)—for example, the attack on Washington DC’s surveillance system in 2017 [17].

## 4. Attacks

**Figure 4:**
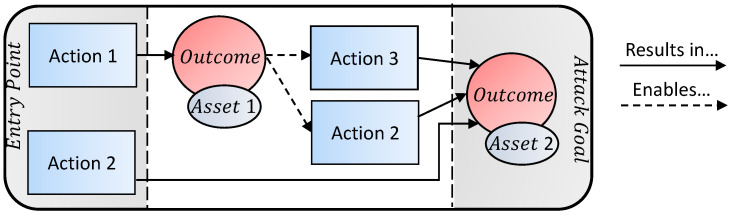
An example of two attack vectors which arrive at the same goal.

There are many different kinds of attacks. Some scenarios involve a single step (e.g., DDoS a VPN link), while others have numerous steps (e.g., stealing credentials by sending a phishing email, then installing a malware, and so on). A sequence of attack steps is often referred to as an attack vector. Each step in the vector gives the attacker access to some asset, and the final step in the vector fulfills the attacker’s goal. As an example, Figure 4 illustrates the flow of two different attack vectors which arrive at the same goal. Here, both Actions 1 and 2 give the attacker access to the surveillance system. However, Action 1 requires addtional attacks to achieve the attack goal as opposed to Action 2 which achieves the goal directly. For example, if the goal were to disable all cameras in the surveillance system, then Action 1 might be ‘brute-force camera credentials’ which results in the outcome ’compromised camera’ giving the attacker control over the asset ’camera’. However, to disable all cameras, the attacker must then attack the DVR (Action 3 or DoS the other cameras (Action 4). On the other hand, the attacker could have achieved his goal from the outset by launching a DDoS attack on the DVR (Action 2) if the attacker owns a botnet.

In summary, an attack vector is one possible route for the attacker to obtain his or her goal. However, not every route is viable considering the attacker’s resources (e.g., tools, physical/remote access, ...), experience, and knowledge of the targeted surveillance network.

To understand an attack vector, one needs to investigate the following aspects:

**Threat** **Agent/Actor.** The person, device, or code
  which performs an attack step on behalf of the attacker.
**Threat** **Action.** The malicious activity
  which an agent can perform at each step (access, misuse, modify, etc.)
**Threat** **Consequence/Outcome.** What the attacker obtains
  at the successful completion of an attack step.
**Attack** **Goal.** The ultimate outcome
  which the attacker is trying to achieve (at the end of the attack vector).

We will now discuss each of these aspects with regard to the surveillance system.

### 4.1. Threat Agents

We identify the following relevant threat agents/actors:

1. **Hacker**—An individual who is experienced at exploiting computer vulnerabilities, whose unauthorized activities violate the system’s security policies. A Hacker can be in a remote location (i.e., the Internet) or in close proximity to the physical network.
2. **Network Host**—A computer connected to the system’s network which is executing malicious code. The computer can be an IP-camera, DVR, or any programmable device in the network. A network host can become a threat actor via local exploitation (the un/intentional instillation of malware—social engineering and insiders), remote exploitation (e.g., exploit a web server vulnerability or an open telnet server), or a supply chain attack [18].
3. **Insider**—An authorized user of the system who is the attacker or colluding with the attacker [19]. The insider may be a regular user (e.g., security officer), an IT support member, or even the system’s administrator. An insider may directly perform the entire attack, or enable a portion of an attack vector by installing malware, changing access permissions, etc.

### 4.2. Threat Actions

We identify the following assaults that a threat agent can perform on the system.

#### 4.2.1. Performing Code Injection

Code injection is an exploitation of improper parsing of an input which results in the input being executed as code. A threat agent may perform a code injection to reveal sensitive information or install some malware. One example is the cross-site request forgery (CSRF) flaw which can be used to add a secondary administrator account on some cameras [20] (for example, CVE-2018-7524, CVE-2018-7512). Another example is the SQL injection attack that has affected Geutebruck G-Cam/EFD-2250 cameras (CVE-2018-7528). Finally, stack-based buffer overflow vulnerabilities have been exploited, and have been discovered in IP-cameras and DVRs (CVE-2017-16725).

Some cameras run local HTTP web xservers to provide users with a convenient configuration interface. However, these servers may be outdated and vulnerable to attack, such as the infamous Heartbleed vulnerability in OpenSSL. Another example is the Sony surveillance camera IPELA series, where parsing vulnerabilities can be exploited to perform a buffer overflow attack via a simple HTTP post message (CVE-2018-3937/8). Other attacks on web servers found on IP-cameras include directory traversals and cgi-bin script exposures. Crafted URLs sent to the server can cause directory traversals which may reveal administrator and Wi-Fi credentials (CVE-2013-2560). Sending various inputs to exposed cgi-bin script URLs can enable live video feeds and enable telnet communications [21].

Even if the traffic is encrypted, the software may be using and old protocol or may be vulnerable to downgrade attacks. For example, Poodle [22] and Beast [23] are attacks which intercept the initial handshake and trick the server into downgrading the encryption to a vulnerable or obsolete exploitable version.

#### 4.2.2. Manipulating/Observing Traffic

A threat agent may manipulate, reroute, or observe network traffic. For example, an agent may (1) perform a man-in-the-middle (MitM) attack in the local network, and then (2) freeze a video image or inject it into a live feed. For the MitM attack, the attacker could reroute traffic through him via ARP poisoning, DHCP/DNS spoofing. For injection, the tool VideoJak may be used to exploit unencrypted video streams using the RTSP or RTP protocols. These protocols are commonly used in video surveillance systems, and may be left unencrypted if found in a PCC deployment.

In the case of traffic observation, an agent may be able to observe video content. In [24], the authors succeeded in extracting JPEG images generated by NetCam IP Camera by sniffing the network traffic. Furthermore, even when the video stream is encrypted, the video footage can be inferred by observing the stream’s bandwidth patterns [25]. This is due to how video codecs (such as H.264) compress motion between frames, and how clients buffer content. Moreover, observing traffic can also reveal network topological information from universal plug and play (UPnP) traffic, and credentials may be revealed as plain text in HTTP traffic (e.g., the DVR in CVE-2017-15290).

At DEFCON’17, VIPER Lab presented (https://youtu.be/XLsoEZzHqjE) [26] an IP video surveillance security assessment tool called VideoJak (part of UCSniff). The tool can be used to intercept Real-time Transport Protocol (RTP) video streams and perform various attacks such as video injection, video looping, and several others. It accomplishes this by (1) performing a man in the middle attack (ARP poisoning), (2) stripping the content from the RTP feed, and (3) replacing the content while updating the CRC.

#### 4.2.3. Exfiltrating Information

Cameras can be exploited to exfiltrate information for an attacker [11]. For example, a malware contained within an isolated network can blink an LED light in view of the camera which is connected to the Internet. By modulating the blinking pattern, the attacker can exfiltrate some stolen information (e.g., user credentials) to a remote location. This has been demonstrated with hard-drives, monitors, and networking equipment [27].

#### 4.2.4. Flooding and Disrupting

A threat agent may prevent access to a service or data by flooding the network with packets, or sending crafted traffic to a network application. A classic DoS attack is to flood a DVR or Camera until the server’s resources are depleted and all new (and sometimes existing) sessions are blocked (e.g., CVE-2019-6973). For example, using the hping3 tool [28], a TCP SYN flood can disable a web server (e.g., CVE-2018-9158) and a UDP flood can overload a network interface. For VPN tunnels, an attacker can disconnect all cameras by flooding the VPN gateway (visible to the Internet). We verified this attack on a Cisco VPN Router by sending numerous key exchange requests using the ISKAMP protocol—resulting in a loss of connection to all cameras. Furthermore, an SSDP amplification attack [29] can be used to overload a DVR. In this attack, the agent causes the cameras to spam the DVR with large amounts of UPnP meta-data by sending requests using the DVR’s IP address.

IP-cameras are often susceptible to these attacks because they are typically resource limited devices. For example, some cameras can only support up to 80 concurrent HTTP connections, which can easily be consumed. Another example is an SSL regeneration attack where the agent repeatedly requests key renegotiations which overloads the device’s CPU. This attack is successful because it is much harder for the server to process the messages than it is for the attacker to produce them.

Other DoS attacks can be accomplished by exploiting bugs and vulnerabilities. For example, a camera can be crashed by sending large HTTP POST requests (CVE-2018-6479), and a VPN router can be forced to drop all connections due to crafted packets (CVE-2014-0674 and CVE-2016-6466).

#### 4.2.5. Scanning and Reconnaissance

A threat agent may perform a network scan to learn the topology, assets, open network ports, and services available for potential exploitation. Off the shelf tools such as NMAP can be used to map the network and reveal information about its hosts. An agent may also elicit responses from web services to reveal version information, and perform fuzzing attacks on exposed web interfaces to find potential vulnerabilities. Fuzzing is typically performed off-site since it is easy to detect.

#### 4.2.6. Exploiting a Misconfiguration

A threat agent may utilize a misconfiguration to install malware or gain access to sensitive data. Example misconfigurations include leaving default credentials, exposed services (e.g., Telnet), and improper access control rules. A misconfiguration can be caused by a user of the system or even the manufacturer. For example, manufactures often leave developer credentials and open network ports in their final releases [24]. We have discovered this to be true in our surveillance testbed which was using high-end Sony Surveillance Cameras [30].

#### 4.2.7. Performing a Brute-Force Attack

A Brute-Force attack is the attempt of guessing a correct input by trying many possible options. Brute-Force attacks can be used to reveal user credentials such as user names and passwords. These attacks can be mitigated by limiting the number of failed logins allowed per minute. However, in some cases, camera manufactures do not implement this security feature. To arrive at a solution quickly, a dictionary of common passwords may be used as a guessing pool. For example, the *Mirai* malware propagated to other devices by connecting via Telnet using a dictionary of 62 common credentials used by cameras, DVRs, and IoTs alike [4]. Another example is the *Remaiten* and *Aidra* malware which compromises cameras and other IoT devices using a similar approach [12]. In addition, the FOSCAM camera was targeted in the same attack since it had no protection against Brute-Forcing [20] while the password was limited to 12 characters only.

#### 4.2.8. Social Engineering

Social Engineering (SE) refers to psychological manipulation of a person which causes him/her to perform an action on behalf of the attacker [31]. Common SE attacks include phishing emails and baiting. In phishing, the threat agent sends a message (email, SMS, etc) disguised as a trustworthy source, in an attempt to get the receiver to install some malware, or ultimately reveal user credentials. In baiting, the threat agent plants a multimedia device (e.g., USB drive or microSD card) loaded with malware. The victim then unwittingly plugs it into his machine which infects it. This can be accomplished with free tools such as [32] and those bundled with Kali Linux.

#### 4.2.9. Physical Access

Physical access is where a threat agent performs an attack which requires direct physical contact with the system. For example, installing a wiretap, backdoor device, accessing a terminal in the server room, flashing a camera’s firmware, obstructing the camera’s view, or simply cutting a wire.

#### 4.2.10. Supply Chain

A supply chain attack is where a threat agent modifies the device (e.g., camera or router) by installing a rootkit or hardware-based spyware during the manufacturing process [18]. Although it is very difficult to accomplish, a successful supply chain attack can provide an attacker with complete control over the device as well as access to the local network.

#### 4.2.11. Reverse Engineering

A threat agent may learn the target device’s credentials or vulnerabilities by the use of reverse engineering (RE) [33]. Reverse engineering is typically performed off-site using the same hardware/software used by the victim. RE is the process of analyzing compiled code or hardware to identify system’s components and their interrelationships. During this process, vulnerabilities and even hard-coded credentials can be discovered.

One approach is to analyze the pre-compiled firmware provided by the manufacturer. In a Black Hat lecture [34], the authors focused on IP cameras which face the Internet and analyze them through firmware images supplied by the camera’s vendors. The authors found zero-day vulnerabilities in digital surveillance equipment from various firms including D-Link Corp., Cisco Systems, Linksys, TRENDnet, and more with the use of existing tools. The analysis revealed serious security vulnerabilities such as administrative passwords, remote code execution vulnerabilities, and more. Another case was found in Sony’s IPELA surveillance camera series. By performing RE on the firmware, researchers from Sec Consult found a backdoor via two hard coded root level credentials. These backdoors have also been discovered in other cameras and DVRs (In CVE-2018-5723 and CVE-2017-6432). The hard-coding of credentials may occur intentionally, or by mistake (e.g., a developer forgot to remove the credentials after testing).

Another approach of RE is to interface with the device via its Universal Asynchronous Receiver/Transmitter (UART) ports. These ports are typically inside the device’s casing, and used by the manufacturer for debugging purposes. UART ports can be used to expose vulneabilites, gain access to the firmware, run foreign applications, extract sensitive information, or upload custom firmware for further analysis. In [35], the authors analyzed several IoT devices including IP cameras, baby monitors thermostats, and doorbells for vulnerabilities. The authors presented a generic workflow in order to gain access to the software of IoT devices, run foreign applications, and extract secret information (i.e., credentials) with the UART ports. In [36], the authors reveal that many DVRs are vulnerable to an unauthenticated login disclosure and unauthenticated command injection via UART. In [37], researchers connected to the UART and updated the firmware with code to find that the camera was running a vulnerable OpenSSL version (i.e., heartbleed) and discovered other vulnerabilities leading to remote code execution.

#### 4.2.12. Adversarial Machine Learning

Video surveillance requires either a manual or automated way of reviewing the video content for events—for example, detecting live intrusions or locating suspects. Therefore, the domain of video analytics has been applied to minimize the human efforts in this task. In the case of large deployments, such as China’s state surveillance system [38], automated methods are required. Some automated technologies include facial recognition [39,40], event detection [41,42,43], and object tracking [44,45,46,47]. However, since most of these technologies rely on machine learning, they are susceptible to adversarial attacks [10]. An adversarial attack is where a machine learning model is abused by either (1) poisoning the model during training so that the mode will behave according to the attacker’s will, (2) crafting an input which will yield an unexpected output, or (3) learning the training data or the model itself by observing the input–output relationship. Adversarial attacks on these technologies mean that an attacker may be able to evade detection, falsify the recognition of an object, or even cause a DoS attack by raising the technology’s false positive rate.

A good example of an adversarial attack on Surveillance systems is the work done in [48]. There the authors generated colorful glasses rims, which, when worn, alters the identity of the individual in the perspective of the deep learning classifier monitoring the imagery. This attack can be used to not only evade detection, but impersonate individuals as well. Another attack on these systems is a DoS attack where the attacker spams the imagery with false positives—for example, by wearing clothes with crafted license plate images to overload traffic cams [49].

Another good example is where the attacker crafts adversarial images which are designed to consume significant resources [14]. An attacker can cause a DoS attack by placing these ‘sponge’ samples in view of the camera to either (1) slow down the device’s processor until it becomes unresponsive or (2) depleting the battery of remote cameras.

Another example is where AI-based surveillance systems setup to measure traffic [50,51,52] can be fooled to reporting traffic jams or no traffic. This would give the attacker the ability to shape traffic to his needs, cause havoc, or block emergency routes to hospitals as an act of terrorism.

We suggest that an attacker may try to spam the system with millions of false alarms, burying important alerts and notifications from the response team’s view. This can be accomplished by crafting adversarial images which contain the thousands of patterns that trigger the object detector. For example, a single picture containing numerous imperceivable patterns of weapons or faces.

### 4.3. Threat Consequence

The success of an assault during an attack step provides the attacker with new capabilities—for example, access to new assets, the ability to run code, and the ability to perform new attacks. We identify the following as the primary threat consequences:

#### 4.3.1. Privilege Escalation

An attacker may receive new credentials or execute code in a way that provides access to previously restricted assets. This escalation can be used to gather information, un/install software, en/disable a protection mechanism, etc. For example, an unprotected web facing CGI method can give an unauthenticated user the ability to bypass the login screen and access the webcam contents including: live video stream, configuration files with all the passwords, system information, and much more (CVE-2017-17101). Another example is CVE-2017-6432 where one can inject new users into DVR management traffic via a MitM attack.

#### 4.3.2. Access to Video Footage

The attacker may be able to watch/download live or pre-recorded video footage. Compared to compromising other IoT devices, this results in a significant privacy violation. The attacker could use the content to track people, observe their behaviors, find where valuables are stored, shoulder-surf to steal credentials, determine when to commit a crime, or blackmail an individual. Another concern is that the attacker will alter the contents to plant false evidence such as a prerecorded video loop, or use deep learning to insert an individual performing an activity (a.k.a., a deepfake [53]), cover up an on-going crime, or permanently delete footage.

In some cases, an attacker may get implicit access to the video content through side-channel attacks. For example, video compression algorithms, such as H264, only send data when regions of the frames change. As a result, the bandwidth of the channel fluctuates in correlation to the motion in the camera’s field of view. The authors of [54] demonstrate this concept on surveillance cameras using the CUMSUM algorithm on the data rates of the encrypted network traffic. CUSUM is a nonparametric algorithm which can detect anomalies in time series data.

#### 4.3.3. Arbitrary Code Execution (Ace)

A significant security threat which enables an attacker to execute any command on a target machine or within a target process. As a result, the attacker can perform privilege escalation, install malware, steal data, and perform other malicious tasks. ACE vulnerabilities have been discovered on IP cameras, DVRs, and VPN routers (for example: CVE-2018-6414, CVE-2018-9156, CVE-2018-9157, CVE-2018-7532, CVE-2018-7512, CVE-2015-8039, CVE-2018-0125, CVE-2017-3882).

#### 4.3.4. Installation of Malware

The attacker may be able to install and execute his own process on a target device. This software is referred to as malware: malicious code designed to damage a computer with malicious intent. Types of malware include worms, trojan horses, viruses, spyware, scarewares, launchers, ransomware, adware, and rootkits. Malware can be used to steal sensitive data, encrypt or delete user data, harm the device, mine crypto currencies, add the device to botnet, or act as a pivot point for lateral movement through the victim’s network.

#### 4.3.5. Lateral Movement

An attacker may gain a stronger foothold in the surveillance system, and achieve the ability to reach previously inaccessible assets. The attacker may also be able to reach other systems and infect user devices connected to the system.

#### 4.3.6. Man in the Middle (MiTM)

An attacker may be able to covertly observe and manipulate traffic between two or more endpoints. An MitM can harm the confidentiality, integrity, and availability (CIA triad) of the system. For example, the MitM can eavesdrop, manipulate, craft, or drop network traffic.

#### 4.3.7. Denial-of-Service (DoS)

An attacker may be able to affect the availability of a service, data, or resource. If the attacker has compromised cameras or the DVR, then the attacker can cause a camera to stop transmitting video content, delete historic content, block access to the DVR, or cause a VPN link to fail (e.g., CVE-2017-3882 and CVE-2016-6466). As a result, a crime may be accomplished on the premises without digital evidence. An attacker may also be able to evade detection without raising any alerts in the DVR. This can be accomplished via a video injection attack or an adversarial machine learning attack.

With regard to DDoS attacks: an attacker may target the surveillance network with a remote botnet. In this case, the consequence of not filtering the traffic is a DoS to the system. We also note that, in the case where the system itself is infected with a botnet, and is then used to launch a remote DDoS attack, the consequence may still be a DoS to the local system since there will be congestion and the ISP may block the system from network access. An example of surveillance systems being used to launch a devastating DDoS attack is the Mirai botnet from 2016 [55].

#### 4.3.8. Access to an Isolated Network

In some cases, the DVR is connected to the Internet (POC or VCC) and is also connected to a network which is supposedly isolated from the Internet (e.g., airports, hospitals, factories, etc.). By compromising the DVR or one of the cameras, the attacker can perform lateral movement into the isolated network. For example, two researchers hacked into a Google office building’s air-conditioning system portal and then gained access to the internal network [56].

#### 4.3.9. Covert Exfiltration Channel

Some surveillance systems are air-gapped (not connected to any other network) as a security measure. Often, these systems are connected to other air-gapped networks (e.g., military installations and airports). An attacker who has infected the air-gapped network with a malware can exploit the surveillance cameras as a means to covertly (1) exfiltrate data from the network such as passwords and documents, and (2) receive commands from the attacker such as ‘start attack’ and ‘self destruct’. An example of this can be seen in [57]. There the authors show how an attacker can use the infra-red (IR) night vision emitters and receivers of a surveillance camera to send and receive optical messages over an air-gap. The malware receives data by monitoring the night vision video feed in a MitM attack and sends data by exploiting the camera’s IR illumination API via the local network. Here, the channel is covert because IR is outside of the human visible spectrum.

### 4.4. Example Attack Vectors

In this section, we provide example attack vectors for different scenarios. Although there are many possible attack vectors, we will illustrate a small sample of common vectors used to attack IP-based surveillance systems. For the illustrations, we use the template presented in Figure 4.

#### 4.4.1. Unauthorized Video Monitoring

**Figure 5:**
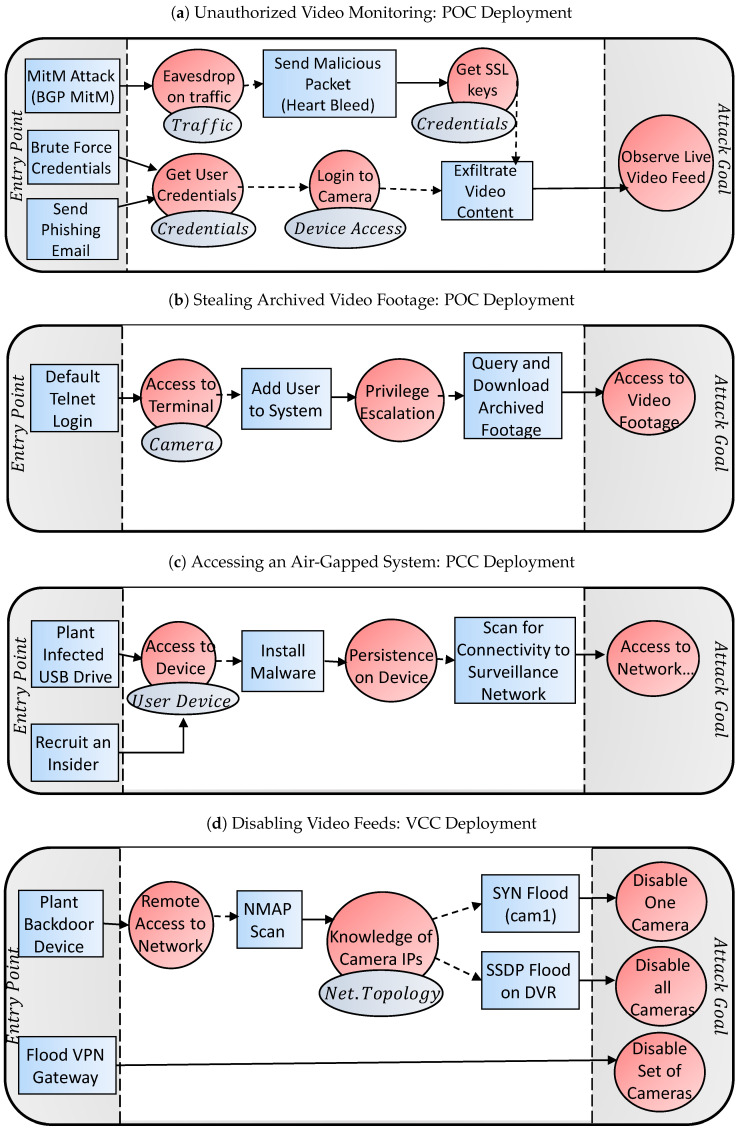
Example attack vectors on IP-based surveillance camera systems deployed with POC, PCC, and VCC topologies.

Consider an attacker who wants to view the video footage of a POC deployment with encrypted traffic. In Figure 5a, a few potential attack vectors are illustrated. A state actor may perform a BGP MitM routing attack, and cause all of the video surveillance traffic to pass through them first. This would give him access to the cameras’ traffic enabling him to eavesdrop on the camera–server communications. Next, the attacker may exploit the Heartbleed vulnerability to get the SSL cryptographic keys and then decrypt the video traffic. A simpler way might be to get the camera’s or DVR’s login credentials by performing a brute-force login attack, or to send phishing emails to users of the system to have them unwittingly reveal their credentials. Once the credentials have been obtained, the attacker can access the camera and observe the live video feeds.

#### 4.4.2. Stealing Archived Video Footage

Another scenario is the case where an attacker wants to blackmail an individual by obtaining sensitive video footage. In Figure 5d, we illustrate one possible attack vector where an agent gains access to the DVR’s terminal by performing a dictionary brute-force login attack on one of the camera’s telnet servers. Having access to a camera’s terminal prompt, the attacker will add a user to the system giving himself elevated privileges to query the DVR for archived footage (e.g., using RTSP replay commands).

#### 4.4.3. Accessing an Air-Gapped System

In the case of a PCC deployment, direct access from the Internet is impossible. However, this does not mean that the network is impervious to infiltration. In Figure 5c, we illustrate how an attacker can install malware on one of the user’s devices, such as a viewing terminal (tablet, console, etc.) This can be accomplished by surreptitiously placing an infected USB drive in the area, or by recruiting an insider. Once the malware is installed by the threat agent, the malware will scan the surveillance network to identify all of the assets. At this point, the malware may perform automated actions designed by the attacker (e.g., disable camera at a certain time) or it may communicate with the attacker directly over the air-gap via bridgeware [13].

#### 4.4.4. Disabling Video Feeds

An attacker can disable video feeds in many different ways. Let’s assume that the target system has a VCC deployment, so access is either physical or via a VPN gateway. In Figure 5b, we show how an attacker can disable one, all, or a subset of cameras in this scenario. First, an attacker may plant a backdoor device to gain remote entry. For example, the attacker may arrive under the pretext of a repairman and secretively connect a Raspberry Pi to the network, and then remotely connecting to the Pi’s Wi-Fi access point from the parking lot. Next, the attacker will have the Pi scan the network to reveal the IP addresses of the cameras and the DVR. Finally, single cameras can be disabled via a TCP SYN flooding attack, or all cameras can be disabled by exploiting a potential SSP flood vulnerability in the DVR. Alternatively, instead of coming locally to plant a backdoor device, the attacker can attack remotely by performing a flood attack (e.g., ISAKMP flood) on the network’s site-to-site VPN gateway. As a result, the set of cameras on one side of the VPN tunnel will be disconnected from the DVR on the other side of the tunnel.

## 5. Countermeasures and Best Practices

In the following section, we review existing countermeasures and best practices which can be used to protect modern surveillance systems.

### 5.1. Intrusion Detection and Prevention Systems

Basic cyber defense should be considered in every computer network. For example, to detect and prevent malware infections, anti-virus software should be installed on the user terminals and DVRs. In non-distributed POC topologies, a strict firewall should be deployed to pass the minimal network traffic required to use the system (e.g., block telnet, ICMP ‘ping’ packets, etc.).

In case the adversary evades the firewall, a network intrusion detection system (NIDS) can be used to detect malicious traffic patterns. In this case, free rule-based NIDS, such as Snort and Suricata, or commercial software can be used.

In [58], the authors propose a lightweight NIDS based on an ensemble of autoencoder neural networks, and evaluate it on a video surveillance system. Kitsune uses incremental statistics to track millions for network channels and then uses these summaries to extract a feature vector for every packet. The feature vector captures a snapshot of the network in the context of the given packet. The anomaly detection model (KitNET) is trained in real time and on site. The model uses autoencoders to detect anomalies. This is accomplished by training the autoencoder to recontruct feature vectors of benign traffic, and then flagging packets whose reconstruction errors are statistically high. KitNET is comprised of an ensemble of small autoencoders, where each one covers a different correlated subspace (set of features), and a single autoencoder which monitors the ensembles’ recontruction errors to cover cross-subspace anomalies. The authors evaluated Kitsune on a commercial IP-based surveillance system and successfully detected two types of reconnaissance attacks, three types of man-in-the-middle attacks, and three types of DoS attacks.

In [59], the authors use a similar model as Kitsune except that the anomalies are detected using the Local Outlier Factor measure (LOF). To evaluate their model, the authors used a number of surveillance cameras and other IoTs and then infected the network with botnet malware such as Mirai.

#### 5.1.1. Configurations and Encryption

One should carefully review the configurations of the cameras, routers, terminals, and DVR. For example, weak or default passwords should be changed, and different passwords should be used among different devices if possible. Moreover, APIs and other similar features should be disabled if not needed. One should also periodically check for new CVEs that the software/firmware of all devices are up to date.

It is also important to enable secure communication wherever possible and not just on the video stream itself [60]. This is because an attacker would still be able to hijack a video stream (e.g., redirect or pause video in an RTSP stream) or compromise the DVR through leaked credentials. We have also noted that several vendors of DVR software use self-signed SSL certificates (a common default setting). This is a significant risk and should be corrected since it enables an attacker to perform an SSL man in the middle redirection attack [61].

To ensure the integrity of the video content, digital watermarks (DW) can be used [45,62,63]. A DW is a subtle signal hidden within the imagery (pixels) which is corrupted if the image is tampered with. In this way, the viewer can verify that every frame is legitimate. The advantage of using DWs is that they do not require changes to the video or networking protocols; however, they may add noise to the image and get corrupted in the presence of video compression.

#### 5.1.2. Restrict Physical Access

The most basic perimeter defense is to restrict physical access to the system’s assets. If possible, wiring should not pass through public areas, all networking equipment (switches, routers, etc.) should be protected under lock-and-key, and access to the system should be managed, logged, and monitored.

#### 5.1.3. Defense against DoS Attacks

There are many protocols and vulnerabilities that can be abused to perform a DoS attack. As a result, there are many different defense mechanisms which can be deployed. Good protection involves the following steps: (1) detect the attack’s initiation, (2) select the malicious/harmful packets, and (3) filter/log the detected packets. For the attack detection, machine learning and statistical methods can be used such as lightweight anomaly detection and many more [64,65,66,67].

#### 5.1.4. Defense against MitM Attacks

Proper encryption should be used to prevent eavesdropping and packet manipulation (e.g., injecting video) as a result of a MitM attack. However, sometimes vulnerabilities are discovered in encryption protocols, and systems may be misconfigured. Therefore, as an additional line of defense, additional methods can be deployed. To detect tampering (video injection), one can reference time according to shadow positions [68,69]. However, this method only works in limited circumstances. Another option is to measure Electric Network Frequency (ENF) signals as a natural time-stamp in indoor locations [70]. A more common approach to verifying image integrity is *watermarking* [62] as described above.

Another approach towards mitigating data tampering is to perform data provenance (proving the source of the data). For example, it has been shown that every camera’s imaging sensor has a unique noise pattern that can be used to identify the source device by its fingerprint [71]. This can be used to identify injected video footage at the end-point. In [72], the authors used a deep convolutional neural network to extract forensic patches from the target video and then assess if the imagery came from the same device using a similarity network. Although the sampling of these fingerprints has been done in the past on images, this work was performed on videos which are often highly compressed and thus leave less evidence. Another example of data provenance was proposed in [73]. There the authors offered a method for tracking the source of surveillance cameras in an IP-based CCTV system data end to end using a blockchain distributed ledger. To protect against data injection attacks in surveillance networks, the authors of [74] propose a means for increasing the robustness of the system by adding trusted nodes. The authors show that there is a practical trade-off between the number of trusted nodes (cost) and the system’s resilience to the attacks.

Another approach is to actively search for the presence of eavesdroppers with the ability to manipulate traffic. In [75], the authors perform echo analysis by bouncing bursts of packets off hosts in the network to detect additional entities along the way. This approach is analogous to how bats chirp and listen to the impulse response to understand where objects are in the environment.

#### 5.1.5. Defense against Adversarial Machine Learning Attacks

There have been a number of works which offer ways to harden AI against adversarial samples [76]. However, there have been a few which specifically address surveillance systems utilizing the temporal aspect of the video footage to detect tampering.

In [77], the authors propose the use of an LSTM deep neural network to predict the next frame in a surveillance footage. When the predicted next frame does not match actual next frame, then an alert is raised. The authors evaluated their approach on various attacks, such as manipulation of the camera’s angle, focus, and change of scene. In [78], the authors present a method for detecting adversarial attacks against crowd counting. Their approach is to use a network which not only predicts the crowd density but also the scene depth. By doing so, the attacker unintentionally affects the scene depth and the anomaly is detected on that output. In [79], the authors propose AdvIT, a neural network which detects adversarial frames by measuring the temporal consistency of the video. This is accomplished by (1) predicting the current frame using the optical flow from the previous frames, (2) segmenting the predicted and actual current frame using a neural network, and (3) measuring the consistency (cross entropy with the Hadamard product) between the two segmentation maps. In [80], the authors propose a countermeasure against adversarial attacks on the image classifier of a surveillance cyber-physical system. Their approach is to use PCA on a clean dataset to obtain an basis on which to examine new instances by perturbing the axis.

#### 5.1.6. Education

An advanced persistent threats (APT) is a well organized attack on an organization that spans numerous attack steps until the attack goal has been achieved [81]. In an APT, the initial intrusion often comes in the form of a social engineering attack, where an employee is tricked into providing credentials or installing malware. The most effective way of mitigating these initial incursions is to: (1) educate the users of the system of the potential attack vectors, and (2) warn users to be careful of unsolicited messages and requests made under false pretexts.

## 6. Future Work & Conclusions

We have identified two main emerging threats to IP-based video surveillance systems. The first is *adversarial machine learning* (Section 4.2.12). Advanced machine learning techniques, mainly based on deep learning, are being researched and integrated within today’s video surveillance systems for automating various tasks including: weapon detection [82], fire detection [83], in-store shopping [84], face recognition [85], and anomaly detection [86]. In parallel, there has been an increase of research on adversarial machine learning [10] meaning that these systems are vulnerable to attacks [87]. The second emerging threat is how these systems are being infected and recruited into botnets [88], leading to attacks on the internal network (e.g., data exfiltration, spying or using the surveillance system for lateral movement) or on other external networks (e.g., DDoS, SPAM).

New attacks are constantly emerging. As a result, have noted in our review that a recent research trend has been securing surveillance systems has been the use of advanced anomaly detection. With anomaly detection researchers are able to identify man-in-the-middle-attacks [75], video injection, OS fingerprinting, fuzzing and ARP poisoning attacks [58], and DDoS attacks [59]. Although anomaly detection is vulnerable to adversarial machine learning, previous works in the domain have mostly focused on attacking classifiers. The key difference between classifiers and anomaly detectors is that classifiers have a decision boundary built into the model (are closed-world) and anomaly detection models only capture the normal behaviors (are open-world). Therefore, more research is needed in understanding how an attacker can potentially craft a sample which is detected as normal while still achieving his or her goal of something that is abnormal.

Updating the software of such systems is also a challenging task since manufactures are focused on their next product, and in many cases do not have the capability of performing remote patching. Therefore, we believe future research should focus on providing an external continuous protection that can be easily updated with information on newly discovered attacks. One way to collect intelligence on emerging threats to surveillance systems is to use an advanced honeypot system [89]. Moreover, by identifying emerging exploits, administrators can protect their systems before they get infected.

Finally, although in most cases the communication of advanced video surveillance systems is encrypted, the confidentially of entities can be compromised using side channel attacks [90]. A vulnerability of encrypted video streams is that the compression algorithm (video codec) generates more data when there is motion. Researchers have shown how content can be inferred from these encrypted streams by learning/correlating network bandwidth patterns [25]. In [91], the authors showed how to detect what a drone’s surveillance camera is looking at by (1) monitoring the encrypted WiFi video stream, (2) triggering a visual stimulus such as a flashing light, and (3) detecting correlated bandwidth fluctuations in the wireless signal. Therefore, we suggest that future research should address the detection and elimination of these side channels.

### Conclusions

In this article, we have reviewed the security of modern video surveillance systems. First, we presented a security overview of these systems along with their components and their deployments. Using this information, we identified the system’s attack surface comprising of its attack agents, vulnerabilities, actions, and consequences. We then used this information to exemplify several attack vectors. Having described the attacker’s capabilities, we then discussed recent research on countermeasures and best practices which can be implemented to better secure IP-based surveillance systems. Finally, we concluded the review with a discussion on the threat horizon, and suggested future work in this domain.

In summary, this article provided the reader with a greater understanding of the attack surface and recent advances made by both the attackers and defenders over the last ten years. We hope that this information will aid researchers and engineers in securing the surveillance systems of today and tomorrow. 

## Figures and Tables

**Table:** 


(a) t

**Table:** 

		
(b) t	(c) t	(d) t

**Table 1:** The attacks performed on the Commercial Surveillance System testbed.

Attack Type	Attack Name	Tool	Description: The attacker...	Violation	Vector
Recon.	**OS Scan**	Nmap	…scans the network for hosts, and their operating systems, to reveal possible vulnerabilities.	**C**	1
	**Fuzzing**	SFuzz	…searches for vulnerabilities in the camera’s web servers by sending random commands to their cgis.	**C**	3
Man in the Middle	**Video Injection**	Video Jack	…injects a recorded video clip into a live video stream.	**C, I**	1
	**ARP MiTM**	Ettercap	…intercepts all LAN traffic via an ARP poisoning attack.	**C**	1
	**Active Wiretap**	Raspberry Pi 3B	…intercepts all LAN traffic via active wiretap (network bridge) covertly installed on an exposed cable.	**C**	2
Denial of Service	**SSDP Flood**	Saddam	…overloads the DVR by causing cameras to spam the server with UPnP advertisements.	**A**	1
	**SYN DoS**	Hping3	…disables a camera’s video stream by overloading its web server.	**A**	1
	**SSL Renegotiation**	THC	…disables a camera’s video stream by sending many SSL renegotiation packets to the camera.	**A**	1
Malware Botnet	**Mirai**	Telnet	…infects IoT with the Mirai malware by exploiting default credentials, and then scans for new vulnerable victims network.	**C, I**	X
